# Influence of Dietary Inulin on Fecal Microbiota, Cardiometabolic Risk Factors, Eicosanoids, and Oxidative Stress in Rats Fed a High-Fat Diet

**DOI:** 10.3390/foods11244072

**Published:** 2022-12-16

**Authors:** Bernat Miralles-Pérez, Maria Rosa Nogués, Vanessa Sánchez-Martos, Àngels Fortuño-Mar, Sara Ramos-Romero, Josep L. Torres, Julia Ponomarenko, Susana Amézqueta, Xiang Zhang, Marta Romeu

**Affiliations:** 1 Functional Nutrition, Oxidation and Cardiovascular Diseases Research Group (NFOC-SALUT), Pharmacology Unit, Department of Basic Medical Sciences, Universitat Rovira i Virgili, C/ Sant Llorenç 21, E-43201 Reus, Spain; 2 Eldine Patología, C/ Plom 32, E-43006 Tarragona, Spain; 3 Department of Biological Chemistry, Institute of Advanced Chemistry of Catalonia (IQAC-CSIC), C/ Jordi Girona 18–26, E-08034 Barcelona, Spain; 4 Department of Cell Biology, Physiology & Immunology, Faculty of Biology, University of Barcelona, Avd/ Diagonal 643, E-08028 Barcelona, Spain; 5 Centre for Genomic Regulation, The Barcelona Institute of Science and Technology, Universitat Pompeu Fabra (UPF), E-08003 Barcelona, Spain; 6 Departament d’Enginyeria Química i Química Analítica and Institut de Biomedicina (IBUB), Universitat de Barcelona, E-08028 Barcelona, Spain; 7 Department of Chemistry, University of Louisville, 2210 S. Brook Street, Louisville, KY E-40292, USA

**Keywords:** obesity, glucose intolerance, insulin resistance, dyslipidemia, fatty liver, gut microbiota, endogenous antioxidants, lipid peroxidation

## Abstract

The present study examined the influence of inulin on fecal microbiota, cardiometabolic risk factors, eicosanoids, and oxidative stress in rats on a high-fat (HF) diet. Thirty-six male Wistar–Kyoto rats were divided into three dietary groups: standard diet, HF diet, and HF diet + Inulin diet. After 10 weeks, the HF + Inulin diet promoted high dominance of a few bacterial genera including *Blautia* and *Olsenella* in feces while reducing richness, diversity, and rarity compared to the HF diet. These changes in fecal microbiota were accompanied by an increased amount of propionic acid in feces. The HF + Inulin diet decreased cardiometabolic risk factors, decreased the amount of the eicosanoids 11(12)-EET and 15-HETrE in the liver, and decreased oxidative stress in blood compared to the HF diet. In conclusion, increasing consumption of inulin may be a useful nutritional strategy to protect against the onset of obesity and its associated metabolic abnormalities by means of modulation of gut microbiota.

## 1. Introduction

Regular consumption of high-energy-density foods containing high amounts of saturated fat and low amounts of fiber contributes to the onset and development of metabolic abnormalities including visceral obesity, insulin resistance, atherogenic dyslipidemia, increased blood pressure, and ectopic fat accumulation in liver and skeletal muscles [1,2]. When occurring together, the cluster of such metabolic abnormalities, referred to as metabolic syndrome, increases the risk of diabetes and cardiovascular diseases [3].

Rodents fed high-fat (HF) diets without fermentable fiber develop metabolic abnormalities with concomitant alterations in the gut microbiota, as may be generally evidenced by a reduced abundance of Bacteroidetes and increased abundance of Firmicutes compared to their lean counterparts [4,5,6]. Furthermore, altered composition of the gut microbiota due to a HF diet lacking fermentable fiber is associated with gut atrophy [7], decreased production of short-chain fatty acids (SCFAs [e.g., acetic, propionic and butyric acids]) [5], and increased translocation of lipopolysaccharides (LPS) from Gram-negative bacteria [8].

Increased LPS can promote inflammatory response in tissues by binding to toll-like receptor 4 and, as a consequence, can impair the action of insulin [8]. Furthermore, inflammatory stimulus activates phospholipase A_2_ enzymes, which hydrolyze membrane phospholipids and then release fatty acids including those containing 20 carbons such as n3 eicosapentaenoic acid and n6 arachidonic acid [9]. Non-esterified eicosapentaenoic and arachidonic acids can be oxidized by cyclooxygenase (COX), lipoxygenase (LOX), and cytochrome P450 (CYP450) enzymes as well as via a non-enzymatic pathway by reactive oxygen species (ROS), producing autocrine and paracrine signaling lipid mediators referred to as eicosanoids. Generally, eicosanoids derived from n6 arachidonic acid have pro-inflammatory properties, but those derived from n3 eicosapentaenoic acid have lower pro-inflammatory properties or even exert anti-inflammatory effects [10]. Activation of phospholipase A_2_ and inflammation are also related to oxidative stress [11,12], which is defined as the imbalance between oxidants and antioxidants due to increased production of the former (e.g., ROS) or inactivation or depletion of the latter (e.g., superoxide dismutase [SOD], catalase [CAT], glutathione peroxidase [GPx], and reduced glutathione [GSH]) [13]. Oxidative stress can impair redox signaling and/or induce oxidative damage to lipids, proteins, and nucleic acids [13], promoting the development of several pathological conditions including obesity, insulin resistance, low-grade inflammation, and metabolic syndrome [14,15,16].

Dietary strategies for increasing the intake of fiber modify the gut microbiota [17,18], affecting metabolism of the host. Supplementation with fermentable fibers, such as inulin, exhibits greater benefits to health than supplementation with poorly fermentable fibers such as cellulose under HF diet conditions [4,7]. Inulin is a linear fructan composed mainly of β-(2,1) fructosyl-fructose linkages, with a degree of polymerization of two to 60 [19]. It is predominantly found in Jerusalem Artichoke and Chicory and can be used as a fat and sucrose replacer in food products [20]. In contrast to sucrose, inulin cannot be hydrolyzed by intestinal enzymes but can be partially metabolized by microbiota in the cecum and colon, resulting in decreased caloric intake, decreased glycemic and insulinemic responses, and increased production of SCFAs [21,22].

Increased consumption of inulin promotes enhanced gut function and prevents metabolic alterations by means of gut-microbiota-dependent mechanisms [4,7]. Nevertheless, the relationship among inulin, gut microbiota and host’s health is not completely understood [7]. SCFAs, main products of fiber fermentation by gut microbiota, are capable of modulating feed intake and metabolism in adipose tissue and liver of the host, especially inhibiting lipogenesis and promoting fatty acid β-oxidation and thermogenesis [23,24,25]. SCFAs can also promote changes in the expression of elongases and desaturase enzymes, which are involved in producing n3 and n6 long-chain fatty acids, in liver but not in perigonadal adipose tissue [26]. The changes in n3 and n6 polyunsaturated-fatty-acid pathways promoted by consumption of inulin might modify the eicosanoid profile. Furthermore, SCFAs may exhibit anti-inflammatory and antioxidant properties via activation of the nuclear factor-erythroid 2-related factor 2 (Nrf2) pathway [27]. Nevertheless, the effects of partial replacement of sucrose by inulin into HF diets on eicosanoids and oxidative stress remain poorly explored.

For these reasons, the aim of the present study was to examine the influence of inulin as a food ingredient used instead of sucrose in food products rich in saturated fat on fecal microbiota, cardiometabolic risk factors, eicosanoids, and oxidative stress in male Wistar–Kyoto rats.

## 2. Materials and Methods

### 2.1. Ethics Statement

The present study complies with guidelines of the European Union (EU Directive 2010/63/EU) and was approved by the CSIC Bioethics Subcommittee and the authorities of Catalonia (reference number 10090).

### 2.2. Animals and Experimental Design

**Figure 1:**
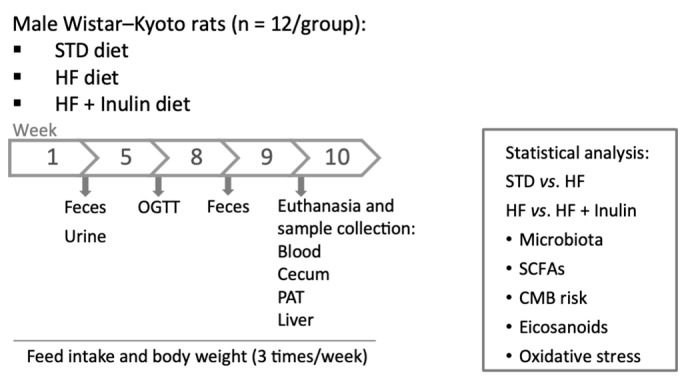
Animal experiment. Abbreviations: STD, standard diet; HF, high-fat diet containing 34% sucrose; HF + Inulin, modified HF diet containing 19% sucrose and 15% inulin; OGTT, oral glucose tolerance tests; PAT, perigonadal adipose tissue; SCFAs, short-chain fatty acids; CMB risk, cardiometabolic risk factors.

Thirty-six male Wistar–Kyoto rats were used (4–5 weeks old; WKY/NHsd, Envigo, Indianapolis, IN, USA). The rats were housed (2–3 rats per Makrolon cage: 425 × 265 × 180 mm) under controlled conditions of humidity (60%), temperature (22 ± 2 °C), and 12 h artificial light/dark cycle with food and water ad libitum. The rats at 9–10 weeks of age were divided into 3 groups (12 rats per group) and fed for 10 weeks one of the following diets: (1) a standard diet (STD group, Teklad Global 14% Protein Rodent Maintenance Diet [2.9 kcal/g]; Envigo, Indianapolis, IN, USA), (2) an HF diet containing 34% sucrose (HF group, TD.08811 45% kcal Fat Diet [4.7 kcal/g], Envigo, Indianapolis, IN, USA), or (3) a modified HF diet containing 19% sucrose and 15% inulin (HF + Inulin group [4.3 kcal/g]). Inulin (Oliggo-Fiber-Inulin Instant, 5–60 fructose units) was provided by Cargill Food Ingredients (Wayzata, MN, USA). The composition of the diets is described in Table S1, and the experimental design is shown in Figure 1.

### 2.3. Feed Intake, Biometric Data, and Feces and Urine Collection

Feed intake and body weight were recorded three times per week. Subsequently, energy intake was estimated using the following conversion factors: 4 kcal/g protein, 9 kcal/g fat, and 4 kcal/g available carbohydrate except for inulin, which used a factor of 1.5 kcal/g inulin [21]. The rats were individually allocated in metabolic cages for 24 h during week 5 to collect fecal and urine samples. Feces were also collected by abdominal massage during week 9 for analysis of gut microbiota composition and measurements of SCFAs. Fecal and urine samples were stored at −80 °C until use. At the end of the study, the body weight gain (final body weight–initial body weight), the adiposity index (perigonadal adipose tissue weight/body weight × 100) and the hepatosomatic index (liver weight/body weight × 100) were calculated.

### 2.4. Sample Processing

The rats were fasted overnight, anesthetized intraperitoneally with ketamine and xylazine (80 and 10 mg/kg body weight, respectively) and sacrificed by exsanguination after 10 weeks. Blood was taken by cardiac puncture into tubes containing anticoagulant heparin or EDTA as appropriate. Plasma was obtained by centrifugation at 850× *g* for 15 min at 4 °C. After the obtaining of plasma, erythrocytes were obtained by washing twice with 154 mM NaCl and centrifugation at 1300× *g* for 5 min at 4 °C. For measurement of glutathione in plasma, proteins were precipitated by adding trichloroacetic acid at a final concentration of 10% (*w/v*). Plasma and erythrocyte samples were stored at −80 °C until use, except one aliquot of erythrocytes that was washed five times with 5 mM Na_2_HPO_4_ and centrifuged at 15,000× *g* for 15 min at 4 °C for obtaining of membranes to assess fluidity.

Cecum, perigonadal adipose tissue, and liver were collected and weighed. After that, the samples were snap-frozen in liquid nitrogen and stored at −80 °C until use, except one part of perigonadal adipose tissue and liver that were fixed for 24 h in 4% formaldehyde for the histological analysis.

One part of the frozen fecal sample was processed by means of a QIAamp^®^ DNA Stool Mini Kit (QIAGEN, Hilden, Germany) for analysis of fecal microbiota. The other part of the frozen fecal sample and the cecal content sample were lyophilized and dissolved in acetonitrile/water (3:7, *v/v*) containing oxalic acid (2.97 g/L) and the internal standard 2-ethylbutyric acid (6.67 mg/L). SCFAs were extracted for 10 min using a rotating mixer. Then, the suspension was centrifuged at 12,880× *g* for 5 min at room temperature, and the supernatant was filtered through a 0.45 µm nylon filter and diluted with acetonitrile/water (3:7, *v/v*) for measurement of SCFAs.

Frozen perigonadal adipose tissue samples were homogenized in 200 mM sodium phosphate buffer (pH 6.25), sonicated for 1 min, and centrifuged at 1000× *g* for 10 min at 4 °C. Then, the supernatant was collected and centrifuged at 129,000× *g* for 1 h at 4 °C. Frozen liver samples were divided into two parts. The first part was homogenized in 200 mM sodium phosphate buffer (pH 6.25) and centrifuged at 129,000× *g* for 1 h at 4 °C for measurement of biomarkers of oxidative stress. The other part of the liver was homogenized in 60% ethanol containing 0.01% butylated hydroxytoluene and spiked with internal standards: 5 ng 15(S)-HETE-d_8_ and PGD_2_-d_9_ (Cayman Chemical, Ann Arbor, MI, USA). After centrifugation at 20,817× *g* for 20 min at 4 °C, liver eicosanoids were extracted using the solid-phase extraction method. Briefly, the supernatant was loaded into a cartridge (Oasis HBL, 3 mL, 60 mg; Waters, Milford, MA, USA), which was pre-conditioned and equilibrated with 3 mL methanol and 3 mL water, respectively. Then, the cartridge was washed with 3 mL 5% methanol, and eicosanoids were eluted by 3 mL acetonitrile/methanol (90:10, *v/v*) onto a tube containing 20 µL 10% glycerol in methanol. After evaporation in a vacuum dryer at 30 °C, the extract was dissolved in 100 µL 40% acetonitrile, centrifuged at 2000× *g* for 5 min at 4 °C, and filtered (Hydrophobic PTFE syringe filter, 0.22 µm; FILTER-LAB, Barcelona, Spain). Tissue samples were stored at −80 °C until use. For measurement of glutathione, proteins from adipose tissue and liver samples were precipitated prior to storage by adding trichloroacetic acid at a final concentration of 10% (*w/v*).

### 2.5. Measurements of Biochemical Parameters

Oral glucose tolerance tests (OGTT) were performed during week 8 on fasting animals as previously described [5]. The area under the curve (AUC) was calculated by means of the Trapezoid rule (AUC, mg/mL per 120 min).

Fasting blood glucose and plasma insulin were measured as described elsewhere [5]. Furthermore, the Homeostatic Assessment Model of Insulin Resistance (HOMA-IR) was calculated as follows: fasting insulin × fasting glucose/22.5 [28].

Blood glycated hemoglobin (HbA1c), plasma triacylglycerol (TAG), total cholesterol (TC), low-density lipoprotein cholesterol (LDLc), high-density lipoprotein cholesterol (HDLc), aspartate aminotransferase (AST), and alanine aminotransferase (ALT) were measured using the corresponding commercial kits (Spinreact, Girona, Spain). Blood hemoglobin (Hb) was measured as described elsewhere [29] for normalization of parameters measured in erythrocytes.

Membrane fluidity of erythrocytes was estimated as previously described [30]. Membrane fluidity was calculated as the inverse value of anisotropy (1/r).

### 2.6. Histological Analysis of Perigonadal Adipose Tissue and Liver Samples

Formalin-fixed perigonadal adipose tissue and liver samples were examined by a single observer as previously described [31] and graded as presented in Tables S2 and S3. The total histological score of perigonadal adipose tissue, ranging from 0 to 11, was the sum of all items detailed in Table S2. The total histological score of liver, ranging from 0 to 10, included the following evaluated items: grade of steatosis, presence of lipogranuloma, presence of microgranuloma, grade of portal chronic inflammation, and grade of sinusoidal dilatation (Table S3).

### 2.7. Analysis of Fecal Microbiota

Total DNA in fecal samples was quantified using a Nanodrop 8000 Spectrophotometer (ThermoScientific, Waltham, MA, USA). All DNA samples were diluted to 5 ng/µL and used to amplify the V3–V4 regions of the 16S ribosomal RNA gene, using the following universal primers in a limited cycle PCR:

Forward primer: 5′ TCG TCG GCA GCG TCA GAT GTG TAT AAG AGA CAG CCT ACG GGN GGC WGC AG

Reverse primer: 5′ GTC TCG TGG GCT CGG AGA TGT GTA TAA GAG ACA GGA CTA CHV GGG TAT CTA ATC C

The above-described primers contain overhangs allowing the addition of full-length Nextera adapters with barcodes for multiplex sequencing in a second PCR step, resulting in sequencing-ready libraries with approximately 450 bp insert sizes. Libraries were eluted in a 20 μL volume and pooled for sequencing. As a control for sequencing and downstream procedures, we also used two DNA samples derived from bacterial mock communities obtained from the ZymoBIOMICS^TM^ Microbial Community DNA Standard, a mixture of genomic DNA of ten microbial strains isolated from pure cultures of eight bacterial and two fungal strains.

Sequencing was performed on an Illumina MiSeq with 2 × 300 bp reads using v3 chemistry with a loading concentration of 10 pM. Sequencing was performed at the Genomics Unit of the Centre for Genomic Regulation, Barcelona.

Sequencing reads were checked for quality using FastQC. 16S amplicons were analyzed using mothur version 1.44.1 [32]. Overlapping pairs of sequence reads were assembled, contigs with more than 4 ambiguities and shorter than 439 bp or larger than 466 bp were discarded, and the remaining contigs were aligned to the reference alignment provided by the SILVA database (version 132) [33] with a k-mer size of 8. Artifacts from the alignment and the contigs with more than 12 homo-polymers (the maximum number found in the reference alignment) were removed. The resulting alignment was simplified by removing the columns containing only gaps and by discarding duplicated sequences. The aligned sequences were then grouped allowing up to 4 mismatches and clusters with only one sequence were removed. Uchime (embedded in the mothur framework) was used to remove chimeras, and the resulting sequences were classified according to the taxonomy into the corresponding operational taxonomic units (OTUs). Undesired lineages such as chloroplast, mitochondria, archaea, eukaryota, and “unknown” were removed. Sequences were then grouped again into OTUs by using the cluster.split command and considering the genus level. Finally, OTUs mapping to the same genus were grouped together. The sequencing error, estimated on the mock samples, was close to zero (<1.0 × 10^−10^).

The 16S rRNA OTUs counts were analyzed using the R package Phyloseq (version 1.36.0.) [34] in R version 4.1.0. Statistical significance of the relative abundances of taxa (>0.01) was evaluated using the Wald test of the DESeq2, version 1.32.0. [35]. In addition, the R package microbiome (version 1.13.12) [36] was used to calculate indexes to estimate within-sample α-diversity, which is the distribution of taxonomic units’ relative abundances in a given sample into a single number, including those for richness (i.e., Chao1 index), evenness (i.e., Pielou, Simpson, and Bulla indexes), dominance (i.e., Simpson, Berger–Parker, Relative, and Gini indexes), rarity (i.e., low abundance index), and diversity (i.e., Shannon and Inverse Simpson indexes). Furthermore, representation of similarities or distances between samples, referred to as between-sample β-diversity (i.e., principal coordinate analysis of unweighted and weighted Unifrac distance), and its significance to differentiate groups (Permanova analysis with Adonis) were calculated using the R packages Phyloseq and vegan (version 2.5.7) [37].

### 2.8. Measurement of Short-Chain Fatty Acids in Feces and Cecal Content

Amounts of SCFAs in fecal and cecal samples were analyzed using a Trace2000 gas chromatograph coupled to a flame ionization detector (ThermoFinnigan, Waltham, MA, USA) equipped with an Innowax 30 m × 530 µm × 1 µm capillary column (Agilent, Santa Clara, CA, USA). Helium was used as carrier gas with a linear velocity of 5 mL/min. Gas chromatography oven temperature was programmed as follows: 80 °C for 1 min, 80 °C to 120 °C at 15 °C/min, hold at 120 °C for 4 min, increase to 130 °C at 5 °C/min, hold at 130 °C for 4 min, increase to 235 °C at 8 °C/min, and hold at 235 °C for 4 min. The detection temperature was 240 °C. Calibration curves were obtained using the internal standard method for six SCFAs (acetic acid, propionic acid, butyric acid, isobutyric acid, valeric acid, and isovaleric acid) in the concentration range of interest. SCFAs were then quantified using the corresponding standard curves. In addition, total SCFAs (sum of individual SCFAs) was calculated.

### 2.9. Measurement of Eicosanoids in Liver

Amounts of non-esterified eicosanoids in the liver were determined as described elsewhere [38] with minor modifications. Liquid chromatography was carried out on an ACQUITY UPLC system coupled to a Xevo TQ-S micro mass spectrometer (Waters, Milford, MA, USA). The chromatographic separation was performed using a BEH C18 column (1.7 µm, 2.1 × 100 mm) protected by a Vanguard pre-column (1.7 µm, 2.1 × 5 mm) purchased from Waters (Milford, MA, USA). The column was maintained at 45 °C and the flow rate was set at 0.6 mL/min. Mobile phases were (A) 0.1% acetic acid and (B) 90:10 acetonitrile/isopropanol (*v/v*). The elution gradient started at 25% B, increased in a linear gradient from 25% to 95% B from 1.00 to 8.00 min, was maintained at 95% B for 0.50 min, and was reconditioned from 8.51 to 10.00 min. The autosampler was kept at 6 °C, and the injection volume was set to 10 µL. The mass spectrometer was operated in negative mode, and the analysis was carried out using a multiple reaction monitoring method (Table S4). Eicosanoids were then quantified using the corresponding standard curves constructed using a series of solutions of compound standards. In addition, the sum of hydroxy-eicosapentaenoic acids derived from n3 eicosapentaenoic acid (HEPEs; 5-HEPE and 11-HEPE), the sum of hydroxy-eicosatetraenoic acids derived from n6 arachidonic acid (HETEs; 5-HETE, 12-HETE, 15-HETE, and 20-HETE), the 5-HEPE/5-HETE ratio, and the sum of prostaglandins derived from n6 arachidonic acid (PG; PGD_2_ and PGE_2_) were calculated.

### 2.10. Measurements of Biomarkers of Oxidative Stress

#### 2.10.1. Plasma Antioxidant Capacity

The oxygen radical absorbance capacity (ORAC) [39] and the ferric reducing ability of plasma (FRAP) assays [40] were performed to assess plasma non-enzymatic antioxidant capacity. 

#### 2.10.2. Antioxidant Enzymes and Glutathione

Activities of the antioxidant enzymes SOD, CAT, GPx and glutathione reductase (GR) were measured in erythrocytes, perigonadal adipose tissue, and liver as previously described [41,42,43].

Amounts of GSH and oxidized glutathione (GSSG) in plasma, perigonadal adipose tissue, and liver were measured as described elsewhere [44]. In addition, the GSSG/GSH ratio was calculated as a marker of the redox state.

#### 2.10.3. End Products of Lipid Peroxidation

Oxidative damage to lipids was assessed using thiobarbituric acid-reactive substances (TBARS) and malondialdehyde (MDA) plus 4-hydroxyalkenals (4-HAE) assays. Amounts of TBARS in plasma, erythrocytes and perigonadal adipose tissue were measured as described elsewhere [45] with some modifications [46]. The amount of non-esterified MDA + 4-HAE in liver was measured as previously described [47].

### 2.11. Statistical Analysis

The statistical analysis was performed using SPSS v.26 software (IBM, Chicago, IL, USA). The results are presented as mean and standard deviation, except for histological results which are presented in frequencies (absolute or %) or median and 25th–75th percentiles. The Shapiro–Wilk test was used for a normality test. Groups were then compared by means of the *t*-test or the Mann–Whitney U test as appropriate (STD vs. HF and HF vs. HF + Inulin). Categorical data were compared with contingency tables using χ^2^ statistics or the Mann–Whitney U test as appropriate. Statistical significance was considered when the *p*-value < 0.05, and a *p*-value between 0.05–0.10 was considered a tendency.

## 3. Results

### 3.1. Feed Intake, Biometric Data, and Feces and Urine

**Figure 2:**
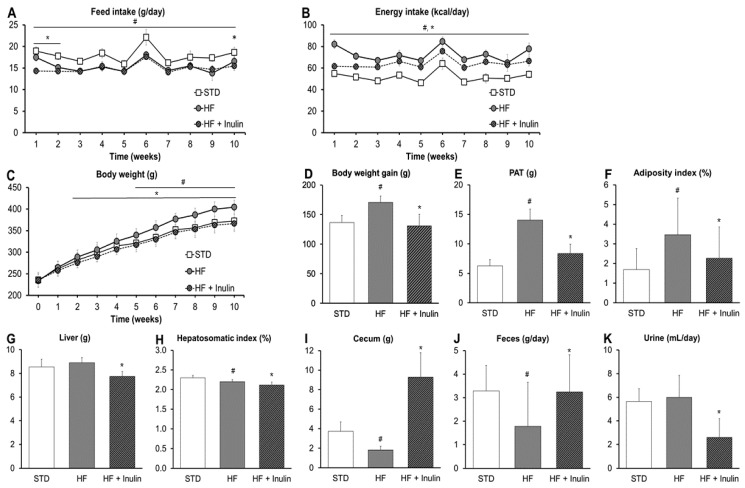
Characteristics of rats. (**A**) Daily feed intake throughout the study. (**B**) Daily energy intake throughout the study. (**C**) Body weight throughout the study. (**D**) Body weight gain (final body weight–initial body weight). (**E**) Perigonadal adipose tissue weight. (**F**) Adiposity index (perigonadal adipose tissue weight/body weight × 100). (**G**) Liver weight. (**H**) Hepatosomatic index (liver weight/body weight × 100). (**I**) Cecum weight. (**J**) Feces in metabolic cages for 24 h during week 5. (**K**) Urine in metabolic cages for 24 h during week 5. Values are expressed as mean with standard deviation. Abbreviations: STD, standard diet; HF, high-fat diet containing 34% sucrose; HF + Inulin, modified HF diet containing 19% sucrose and 15% inulin. *p*-values were calculated using the *t*-test or the non-parametric Mann–Whitney U test. ^#^ *p*-value < 0.05 vs. STD; and * *p*-value < 0.05 vs. HF.

The HF and HF + Inulin diets promoted a decrease in daily feed intake compared to the STD diet throughout the study (Figure 2A). Partial replacement of sucrose by inulin in the HF diet decreased daily feed intake during weeks 1, 2 and 10 (*p*-value < 0.05; Figure 2A). According to the feed intake and energy density of diets, the STD and HF + Inulin groups showed lower energy intake than the HF group (*p*-value < 0.05; Figure 2B).

The HF diet promoted body-weight gain and adiposity gain compared to the STD diet (Figure 2C–F) while reducing values of the hepatosomatic index and cecum weight (Figure 2H,I). The HF + Inulin diet decreased body-weight gain from week 2 and decreased adiposity compared to the HF diet (*p*-value < 0.05; Figure 2C–F). Furthermore, the HF + Inulin diet decreased liver weight and markedly increased cecum weight (*p*-value < 0.05; Figure 2G–I).

The HF diet reduced the content of feces collected in metabolic cages for 24 h during week 5 compared to the STD diet. The HF + Inulin diet increased defecation and decreased excretion of urine compared to the HF diet (*p*-value < 0.05; Figure 2I,J). During the period in metabolic cages, no statistically significant differences in either feed intake or water consumption were found between the two groups under HF diet conditions (g of feed: HF, 13.2 ± 2.6; HF + Inulin, 12.5 ± 2.3. mL of water: HF, 21.4 ± 5.6; HF + Inulin, 22.4 ± 4.7; *p*-value > 0.05).

### 3.2. Biochemical Parameters

**Figure 3:**
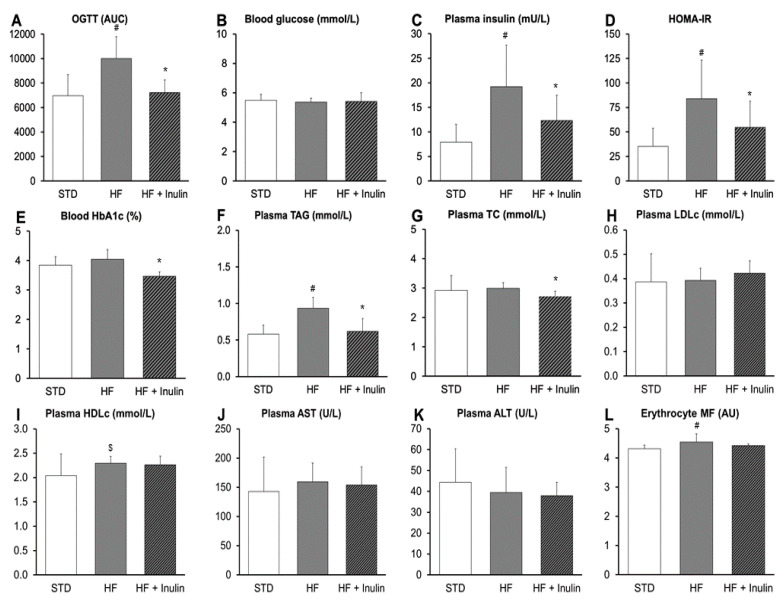
Biochemical parameters of fasting animals. (**A**) Oral glucose tolerance test (OGTT) during week 8. (**B**) Blood glucose. (**C**) Plasma insulin. (**D**) Homeostatic Assessment Model of Insulin Resistance (HOMA-IR, insulin × glucose/22.5). (**E**) Blood glycated hemoglobin (HbA1c). (**F**) Plasma triacylglycerol (TAG). (**G**) Plasma total cholesterol (TC). (**H**) Plasma low-density lipoprotein cholesterol (LDLc). (**I**) Plasma high-density lipoprotein cholesterol (HDLc). (**J**) Plasma aspartate aminotransferase (AST). (**K**) Plasma alanine aminotransferase (ALT). (**L**) Erythrocyte membrane fluidity (MF). Values are expressed as mean with standard deviation. Abbreviations: STD, standard diet; HF, high-fat diet containing 34% sucrose; HF + Inulin, modified HF diet containing 19% sucrose and 15% inulin; AUC, area under the curve (mg glucose/mL blood per 120 min); AU, arbitrary unit. *p*-Values were calculated using the *t*-test or the non-parametric Mann–Whitney U test. ^#^ *p*-value < 0.05 vs. STD; ^$^ *p*-value = 0.075 vs. STD; and * *p*-value < 0.05 vs. HF.

The HF diet promoted oral glucose intolerance, hyperinsulinemia, and increased HOMA-IR compared to the STD diet by the end of the study (Figure 3A,C,D). However, the fasting blood glucose concentration and the percentage of blood HbA1c remained unchanged at the end of the study (Figure 3B,E). The HF + Inulin diet significantly enhanced glucose tolerance and prevented the onset of insulin resistance compared to the HF diet (*p*-value < 0.05; Figure 3A,C,D). Furthermore, the HF + Inulin diet reduced the percentage of blood HbA1c compared to the HF diet (*p*-value < 0.05; Figure 3E), without affecting the fasting blood glucose concentration (*p*-value > 0.05; Figure 3B).

The HF diet promoted an increase in plasma TAG and erythrocyte membrane fluidity compared to the STD diet by the end of the study (Figure 3F,L). The HF + Inulin diet prevented the increase in plasma TAG promoted by the HF diet and even decreased plasma TC (*p*-value < 0.05; Figure 3F,G). No statistically significant differences were observed in either plasma LDLc, plasma HDLc, plasma AST, plasma ALT, or erythrocyte membrane fluidity between the two groups under HF diet conditions (*p*-value > 0.05; Figure 3H–L).

### 3.3. Histological Analysis of Perigonadal Adipose Tissue and Liver Samples

**Figure 4:**
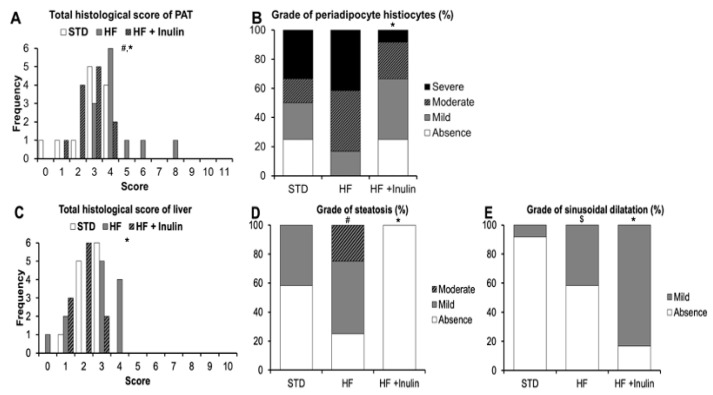
Histological analysis of perigonadal adipose tissue and liver samples. (**A**) Total histological score of perigonadal adipose tissue (PAT) sample. (**B**) Grade of periadipocyte histiocytes. (**C**) Total histological score of liver sample. (**D**) Grade of steatosis in liver. (**E**) Grade of sinusoidal dilatation in liver. Results are expressed in frequencies (absolute or %), *n* = 12 rats/group. Abbreviations: STD, standard diet; HF, high-fat diet; HF + Inulin, modified HF diet containing 15% inulin. *p*-values were calculated using the non-parametric Mann–Whitney U test. ^#^ *p*-value < 0.05 vs. STD; ^$^ *p*-value = 0.065 vs. STD; and * *p*-value < 0.05 vs. HF.

The HF diet promoted a higher total histological score of perigonadal adipose tissue than the STD diet (Figure 4A and Table S2). The most relevant item in the score was the presence of histiocytes. In fact, all rats fed the HF diet showed some grade of histiocytes in the periadipocyte region (Figure 4B and Table S2). The increase in this histological feature was prevented by partial replacement of sucrose by inulin (*p*-value < 0.05; Figure 4A,B and Table S2).

In the liver, the HF diet increased the grade of steatosis in the periportal region and inhibited infiltration of inflammatory cells in the portal region compared to the STD diet (Figure 4D and Table S3) but did not modify the total histological score (Figure 4C). The HF + Inulin diet markedly decreased the accumulation of fat in the liver (*p*-value < 0.05; Figure 4D and Table S3), without affecting the grade of inflammatory infiltrates (*p*-value > 0.05; Table S3). Interestingly, rats fed the HF + Inulin diet showed a higher grade of sinusoidal dilatation than those rats fed the HF diet or the STD diet (*p*-value < 0.05; Figure 4E and Table S3).

### 3.4. Analysis of Fecal Microbiota

The HF diet promoted an increase in the relative abundance of the Proteobacteria phylum compared to the STD diet. The HF + Inulin diet significantly increased the relative abundance of the Actinobacteria phylum and decreased the Firmicutes phylum compared to the HF diet (*p*-value < 0.05).

At the genus level, the HF diet promoted increases in the relative abundances of *Desulfovibrio*, *Escherichia/Shigella*, *Rothia*, *Blautia*, and *Clostridium* XIVa, while promoting decreases in unclassified *Candidatus saccharibacteria*, unclassified *Muribaculaceae*, *Prevotella*, and *Clostridium* IV. The HF + Inulin diet decreased the abundances of *Desulfovibrio*, *Rothia*, unclassified *Firmicutes*, *Lactococcus*, *Ruminococcus*, unclassified *Ruminococcaceae*, unclassified *Lachnospiraceae*, *Clostridium* XIVa, and *Flintibacter*, but increased *Olsenella*, *Blautia*, *Ligilactobacillus*, *Fecalibaculum*, unclassified *Erysipelotrichaceae*, *Bacteroides*, and *Parabacteroides* compared to the HF diet (*p*-value < 0.05).

As far as α-diversity is concerned, the HF diet promoted an increase in evenness (abundance of OTUs) and a decrease in rarity at the phylum level compared to the STD diet. Inclusion of inulin into the HF diet reduced richness (total number of OTUs) at both phylum and genus level compared to the HF diet, while evenness at the phylum level was increased (*p*-value < 0.05). Furthermore, consumption of inulin increased the dominance indexes, reduced the Shannon and Inverse Simpson diversity indexes, and reduced the rarity index at the genus level (*p*-value < 0.05).

As far as β-diversity is concerned, there were no differences between the STD and HF groups at the phylum and genus levels. Consumption of inulin under HF diet conditions induced no effect on β-diversity at the phylum level but markedly affected β-diversity at the genus level compared to both HF and STD groups.

### 3.5. Short-Chain Fatty Acids in Feces and Cecal Content

The HF and HF + Inulin diets decreased the amount of SCFAs especially in feces compared to the STD diet (Table 1). The HF + Inulin diet increased the amounts of acetic, propionic, and butyric acids in feces after 5 weeks compared to the HF diet (*p*-value < 0.05; Table 1). By the end of the study (week 9), only significant differences in propionic acid in feces were found between the HF and HF + Inulin groups (*p*-value < 0.05; Table 1). In cecum, no statistically significant differences were found in the amount of SCFAs between groups (*p*-value > 0.05; Table 1).

### 3.6. Eicosanoids in Liver

The HF and HF + Inulin diets promoted a decrease in the amount of non-esterified eicosanoids derived from n-3 eicosapentaenoic acid (5-HEPE and 11-HEPE) as well as those derived from n-6 arachidonic acid (5-HETE, 20-HETE, 11(12)-EET, 12-HETE, 15-HETE, PGD_2_, and PGE_2_) compared to the STD diet (Table 2). The HF + Inulin diet decreased the amounts of 11(12)-EET derived from n6 arachidonic acid and 15-HETrE derived from n-6 dihomo-γ-linolenic acid compared to the HF diet (*p*-value < 0.05; Table 2). The eicosanoids 15d-PGJ_2_, 17(18)-EpETE, 18-HEPE, LTB_4_, LxA_4_, and PGD_1_ were not detected in liver samples under our experimental conditions.

### 3.7. Biomarkers of Oxidative Stress

The HF diet increased values of ORAC and slightly decreased the amount of liver MDA + HAE compared to the STD diet (Table 3 and Table 4). Both HF and HF + Inulin diets promoted an increase in plasma GSH, erythrocyte TBARS, and liver GPx together with a decrease in adipocyte TBARS compared to the STD diet (Table 3 and Table 4). Furthermore, the two groups under HF diet conditions showed lower erythrocyte and adipocyte GR activity than the group on the STD diet (Table 3 and Table 4). The HF + Inulin diet increased plasma GSH with a concomitant decrease in plasma TBARS compared to the HF diet (*p*-values < 0.05; Table 3).

## 4. Discussion

The present study shows that partial replacement of sucrose by inulin in a HF diet effectively prevented the onset of cardiometabolic risk factors such as body weight gain, perigonadal adipose tissue weight gain, impaired oral glucose tolerance, insulin resistance, hypertriglyceridemia, and slight liver steatosis. These findings agree with the observations of previous studies [4,7,48,49] but contrast with those findings reported by other authors [50]. The discrepancies in the effects of inulin on cardiometabolic risk factors among studies may be attributed to the distinct dose of inulin used in the study of Tan et al. [50] (5% inulin) and those used in our study and in other studies (7−20% inulin) [4,7,48,49]. All together, these findings suggest that inulin dose-dependently reduces the risk for developing obesity and its associated metabolic abnormalities, as previously reported by other authors [51]. Increased intake of fiber is related to reduced feed intake, delayed digestion and decreased absorption of energy [52], which may in part explain the benefits of inulin on biometric and biochemical parameters observed in the present study. However, it has been suggested that the benefits of inulin on adiposity and glucose homeostasis are independent of reduced energy intake, suggesting that modulation of gut microbiota and gut hormones by inulin plays a critical role in the benefits observed [51].

Inclusion of inulin instead of sucrose into the HF diet normalized defection but reduced urine excretion, which could be related to fiber’s properties as a bulking agent to absorb and hold on to water throughout the gut [52]. Once in the cecum and colon, inulin can stimulate the growth of beneficial bacteria. In this sense, we observed that partial replacement of sucrose by inulin in the HF diet increased the weight of the cecum, as previously observed by other authors [7,51,53]. The cecum is the main site for fermentation of inulin in the body [54], and the increase in cecum weight may indicate increased microbial mass [53]. In the present study, consumption of inulin promoted changes in fecal microbiota compared to both the HF and STD groups, as evidenced by α-diversity and β-diversity analysis at the genus level. Interestingly, consumption of inulin under HF diet conditions increased the dominance of few genera, promoting lower richness, diversity, and rarity at the genus level than those observed in animals without inulin. These findings are consistent with the observations reported by other authors [4,55], who considered reduced microbiota diversity as a characteristic of dysbiosis [4]. However, another study found increased richness in mice fed an HF diet after receiving inulin [56]. Other authors showed that inulin exerts no effects on richness [50]. The discrepancies in the effects of inulin on the richness and diversity of fecal microbiota may also be attributed to the distinct dose of inulin used among studies (5% inulin in [50,56] and 10, 15 and 20% inulin in [55], the present study, and [4], respectively). All together, these observations suggest that high doses of inulin may markedly potentiate the growth of certain bacteria whereas low doses promote a diversity of gut microbiota.

Particularly, compared to HF group, rats fed HF + Inulin increased the relative abundance of the genus *Olsenella*, which may explain the increase in the Actinobacteria phylum observed. This agrees with the observations of other authors in mice supplemented with inulin under STD diet conditions [57]. The increase in *Olsonella* observed in the present study was accompanied by a decrease in *Rothia* from the phylum Actinobacteria. Other authors have shown that consumption of inulin at doses of 7% and 20% in the diet of mice increases relative abundance of the Actinobacteria phylum in feces mainly due to an increased abundance of the genus *Bifidobacterium*, which has also been associated with lean phenotype [4,23]. In rats, consumption of inulin at doses of 10 and 25% in the diet increased the abundance of *Bifidobacterium* spp. in cecal content [51]. Nevertheless, the effect of inulin on *Bifidobacterium* was not observed in the present study. This agrees with a previous study in mice fed an STD diet enriched with inulin [58]. Although we found no differences in the Bacteroidetes phylum between the two groups under HF diet conditions, consumption of inulin increased the relative abundance of the genera *Bacteroides* and *Parabacteroides*, as previously observed by other authors [56], while *Prevotella* remained unchanged. The increase in relative abundance of these genera of Bacteroidetes after receiving inulin was accompanied by decreases in the abundances of several genera of Firmicutes, including *Lactococcus*, *Ruminococcus*, *Clostridium* XIVa, and *Flintibacter*. On the other hand, the relative abundances of other genera of Firmicutes including *Blautia*, *Ligilactobacillus*, and *Fecalibaculum* increased after receiving inulin. A similar marked increase in the relative abundance of *Blautia* promoted by inulin in mice was previously reported [58]. In fact, the abundance of *Blautia* is negatively correlated with visceral fat area in humans [59]. In the present study, the HF + Inulin diet significantly reduced the relative abundance of the genus *Desulfovibrio* (Proteobacteria) compared to the HF diet. A decrease in the abundance of *Desulfovibrio* has been also observed in humans with obesity after receiving inulin for three months and was associated with decreased body mass index and body weight [60].

The use of inulin as a substrate for fermentation by gut microbiota favors the production of SCFAs. Consumption of inulin increased the amount of acetic, propionic, and butyric acids in feces after 5 weeks, but only the increase in propionic acid was maintained until the end of the study. Previous studies have also shown that supplementation with inulin increases the amount of SCFAs in feces [4,7,50]. The lack of greater differences in fecal and cecal amounts of SCFAs by the end of the study between the two groups under HF diet conditions may be due to an increased uptake of SCFAs, which has been suggested as a key factor for the benefits of fermentable fibers on the cardiometabolic profile [61].

SCFAs can modulate the host’s energy metabolism by binding to specific G-protein-coupled receptors (GPR) in the gut and in other organs [24,62]. Previous studies have shown that SCFAs derived from the fermentation of inulin promote the secretion of the anorexigenic hormones glucagon-like peptide-1 (GLP-1) and peptide YY (PYY) from the gut, contributing to a reduced food intake [4,51,63]. In the present study, a probable increase in the gut hormones GLP-1 and PYY after receiving inulin may, at least in part, explain why the rats fed the HF + Inulin diet showed a lower feed intake during week 1 and 2 and throughout the study than the rats fed the HF diet and those fed the STD diet, respectively. However, according to Singh et al. [51], differences in feed intake due to reduced preference for a diet rich in fiber compared to a diet poor in fiber between the HF and HF + Inulin groups cannot be excluded. From week 3, the two groups under HF diet conditions showed similar daily feed intakes but significant differences in body weight. Other studies have shown that HF diets increase adipose tissue weight and, as a consequence, increase plasma concentrations of the orexigenic hormone leptin compared to STD diets [64,65]. The increase in body weight, adiposity, and secretion of leptin induced by an HF diet can be prevented by consumption of inulin, as previously reported by other authors [51,63]. Therefore, in the present study, a probable increase in secretion of leptin after 1–2 weeks on an HF diet may explain, at least in part, the comparable values in daily feed intake among rats under HF diet conditions, and the reduced daily feed intake compared to those rats fed an STD diet.

SCFAs can enhance metabolism of lipids by increasing fatty acid β-oxidation and reducing lipogenesis via activation of the AMPK pathway in adipose tissue and liver [23,24,25]. In the present study, partial replacement of sucrose by inulin in the HF diet prevented development of obesity and markedly reduced the accumulation of fat in the liver, which is a major contributor to impaired homeostasis of glucose in both rodents [66] and humans [67]. In agreement with our observations, increased propionic acid has been associated with decreased adiposity and insulin resistance under HF diet conditions [23,26]. The meaning of the slight increase in the grade of sinusoidal dilatation found in the present study after receiving inulin remains unclear. This fact may be related to the high doses of inulin used, but further evaluation is required to identify the causes. No other adverse changes were detected by means of liver histological analysis in rats fed the HF diet + Inulin, and plasma transaminases as markers of liver function were similar among the groups.

Partial replacement of sucrose by inulin in the HF diet reduced the grade of histiocytes in the periadipocyte region of perigonadal adipose tissue samples, without significant changes in inflammatory infiltrates in the liver. A previous study has shown that consumption of inulin by means of GPR43 reduces inflammatory cell infiltration in subcutaneous adipose tissue of mice, but not in perigonadal adipose tissue, and reduces spontaneous release of tumor necrosis factor α in perigonadal adipose tissue [63]. However, it has been suggested that the benefits of inulin on low-grade inflammation and metabolic syndrome are mediated by interleukin-22, but not SCFAs [4]. Other authors have shown that increased production of SCFAs by gut microbiota is not only associated with reduced insulin resistance but also with reduced inflammatory markers including NF-ĸΒ and IL-6 in liver [56]. Distinct stages in the development of disease may explain why we found no significant infiltration of inflammatory cells within the liver under HF diet conditions.

The HF and HF + Inulin diets promoted lower infiltration of inflammatory cells in the portal zone of the liver, amounts of eicosanoids in the liver, and amounts of TBARS in perigonadal adipose tissue than the STD diet. These differences may be, at least in part, explained by the fatty-acid composition of the HF and STD diets. The HF diet provides lower amounts of n6 polyunsaturated fatty acids but higher amounts of saturated fatty acids than the STD diet. Differences in the incorporation of lipids were evidenced in previous studies by a reduced amount of polyunsaturated fatty acids and increased amount of saturated fatty acids in adipose tissue [68] and liver [69] samples after receiving the HF diet. However, a similar experimental model (strain, gender, initial age, initial body weight, diet) showed increased plasma IL-6 after 10 weeks on the HF diet compared to those fed the STD diet and increased lobular inflammation in liver as well as increased IL-6 and PGE2 in plasma after week 24 [5,70]. The apparently contradictory findings between the two studies may indicate that 10 weeks under our HF diet conditions (44% kcal mainly derived from milk fat) is not enough time to effectively promote low-grade inflammation in Wistar–Kyoto rats.

Partial replacement of sucrose by inulin into the HF diet decreased the amount of some non-esterified eicosanoids derived from n6 polyunsaturated fatty acids. To the best of our knowledge, this is the first time that the decline in hepatic eicosanoids after receiving inulin has been reported. Previous studies have shown that consumption of inulin promotes increases in the abundances of n3 α-linolenic acid and n6 linoleic acid together with a decrease in the abundance of n6 dihomo-γ-linolenic acid [49] or arachidonic acid [26] in liver under HF diet conditions, without significant differences in the abundance of eicosapentaenoic acid. The changes in fatty acid profile reported in these previous studies were accompanied by modulation desaturase and elongase enzymes [26,49]. Parent fatty acids were not measured in the present study. However, because the ratio of 5-HEPE to 5-HETE is positively correlated with the ratio of n3 eicosapentaenoic acid to n6 arachidonic acid [71], our findings may suggest a lower abundance of n6 polyunsaturated fatty acids in livers of rats fed the HF + Inulin diet than in the livers of rats fed the HF diet. We cannot exclude differences in fatty acid and eicosanoid profiles partly due to distinct daily feed intake observed between the two groups under HF diet conditions.

The most relevant differences in liver eicosanoids between the two groups under HF diet conditions were found in the amount of 11(12)-EET derived from n6 arachidonic acid via CYP450 enzyme and in the amount of 15-HETrE from n6 dihomo-γ-linolenic acid via LOX enzyme. Consumption of inulin also tended to decrease the amount of HETEs produced by LOX. In line with our findings, amounts of 15-HETrE, 5-HETE, 12-HETE and 15-HETE in plasma are increased in obesity conditions in humans [72,73]. PGE_2_ derived from arachidonic acid via COX enzyme was the only selected and detected eicosanoid that tended to increase after receiving inulin. Since EETs can inhibit COX enzyme [74,75], the decrease in the amount of 11(12)-EET observed may contribute to a slightly increased amount of PGE_2_. Although PGE_2_ is considered a pro-inflammatory lipid mediator, it can exhibit anti-inflammatory properties by reducing the production of cytokine tumor necrosis factor α, inhibiting 5-LOX enzyme involved in producing pro-inflammatory 4-series leukotrienes and 5-HETE, and enhancing 15-LOX enzyme involved in producing pro-resolving lipoxin A_4_ [10]. In fact, plasma PGE_2_ may be negatively correlated with parameters of adiposity, insulin resistance, and fatty liver in healthy young adults [72].

The HF diet exerted no effect on redox state or lipid peroxidation compared to the STD diet. A plausible reason for this is that the HF diet background, with inulin or not, enhanced endogenous antioxidant response compared to the STD diet (i.e., increased plasma GSH and liver GPx). This endogenous antioxidant response under HF diet conditions could be induced to protect against the detrimental effects of increased production of ROS by fatty acid β-oxidation within mitochondria and peroxisomes as previously reported by other authors [16,76,77]. Previous studies have shown that antioxidant enzymes are depleted in advanced stages of disease with a concomitant increase in lipid peroxidation [14,56]. Our findings agree with those reported in other studies [78,79], and the liver antioxidant response under HF diet conditions may be mediated by Nrf2 [80,81]. Both GPx and CAT catalyze the conversion of H_2_O_2_ to H_2_O, but GPx has a higher affinity for H_2_O_2_ than CAT. Therefore, GPx in the cytosolic fraction of liver samples may be activated under subtoxic amounts of H_2_O_2_ mainly released by mitochondria and peroxisomes [77]. Similar activity of GPx observed in perigonadal adipose tissue and liver of rats from the two groups under HF diet conditions may suggest that partial replacement of sucrose by inulin in the HF diet prevented the onset of metabolic abnormalities mainly by reducing lipogenesis and fatty acid uptake rather than increasing fatty acid β-oxidation. Distinct profiles in other parameters of oxidative stress or in other cell compartments, such as mitochondria, cannot be excluded between the two groups under HF diet conditions. In this sense, previous studies have shown that mitochondrial proteins of the liver are especially vulnerable to oxidative damage after an HF diet [82].

Partial replacement of sucrose by inulin in the HF diet decreased oxidative stress in blood but not in tissues. Some studies have shown that supplementation with inulin attenuates oxidative stress in liver of mice fed an HF diet [56] and in serum and amygdala of rats with diabetes [83]. It has been suggested that SCFAs can enhance endogenous antioxidant response via activation of the Nrf2 signaling pathway [27], which agrees with observations by other authors in mice after receiving inulin under HF diet conditions [56]. Distinct stages of disease may explain why parameters of oxidative stress in tissues were unchanged in our study. In this fashion, mice from the study of Du et al. [56] developed liver injury after an HF diet containing 30% by weight of lard for 8 weeks, but this feature was not observed in our study.

Partial replacement of sucrose by inulin in the HF diet tended to decrease values of non-enzymatic antioxidant capacity in plasma. These findings are consistent with previous reports in humans [84] and in rodents [85] on increased non-enzymatic antioxidant capacity in obesity conditions compared to normal-weight conditions. Because a high fraction of FRAP assay represents the antioxidant ability of uric acid [86], our findings may suggest that partial replacement of sucrose by inulin in the HF diet tended to decrease uric acid in plasma. Other authors have reported that HF diets significantly increase plasma concentration of uric acid in advanced stages of disease [85,87] but not in earlier stages [5].

## 5. Conclusions

Increasing consumption of fermentable fiber by means of partial replacement of sucrose by inulin into food products rich in saturated fat may be a useful nutritional strategy to protect against the onset of obesity and its associated metabolic abnormalities including impaired oral glucose tolerance, insulin resistance, hypertriglyceridemia, and liver steatosis. Consumption of inulin induced significant changes in fecal microbiota composition, reducing richness, diversity, and rarity while increasing dominance at the genus level. Furthermore, prevention of metabolic abnormalities promoted by inulin favors a decrease in the amounts of 11(12)-EET and 15-HETrE in the liver, a decrease in the amount of TBARS in plasma, and an increase in the amount of GSH in plasma. Additional studies are needed to determine the biochemical and molecular mechanisms of dietary inulin on cardiometabolic health, including assessment of distinct dosages and its comparison with other types of soluble fibers.

## Figures and Tables

**Table 1:** Short-chain fatty acids in feces and cecal content (dry matter).

	STD	HF	HF + Inulin
Feces			
Week 5			
Acetic acid (mmol/kg)	177 ± 111	63 ± 28 ^#^	144 ± 95 *
Propionic acid (mmol/kg)	18 ± 11	4.2 ± 3.1 ^#^	34 ± 32 *
Butyric acid (mmol/kg)	16 ± 14	3.8 ± 2.8 ^#^	26 ± 26 *
Isobutyric acid (mmol/kg)	1.7 ± 1.0	0.72 ± 0.84 ^#^	0.26 ± 0.14
Valeric acid (mmol/kg)	2.0 ± 1.4	0.75 ± 0.64 ^#^	1.2 ± 1.2
Isovaleric acid (mmol/kg)	2.3 ± 1.4	1.2 ± 1.0 ^#^	0.80 ± 0.69
Total SCFAs	215 ± 130	56 ± 40 ^#^	189 ± 144 *
Week 9			
Acetic acid (mmol/kg)	133 ± 57	29 ± 9 ^#^	38 ± 17
Propionic acid (mmol/kg)	14 ± 8	2.2 ± 1.6 ^#^	5.7 ± 3.6 *
Butyric acid (mmol/kg)	28 ± 24	1.6 ± 1.8 ^#^	0.81 ± 0.75
Isobutyric acid (mmol/kg)	1.3 ± 0.7	0.23 ± 0.21 ^#^	0.15 ± 0.15
Valeric acid (mmol/kg)	1.4 ± 0.7	0.15 ± 0.07 ^#^	0.09 ± 0.06
Isovaleric acid (mmol/kg)	1.2 ± 0.5	0.28 ± 0.24 ^#^	0.32 ± 0.30
Total SCFAs	178 ± 84	31 ± 14 ^#^	40 ± 19
Cecal content			
Acetic acid (mmol/kg)	110 ± 26	99 ± 35	100 ± 36
Propionic acid (mmol/kg)	32 ± 6	28 ± 8	28 ± 9
Butyric acid (mmol/kg)	20 ± 7	18 ± 9	18 ± 6
Isobutyric acid (mmol/kg)	3.0 ± 1.1	2.8 ± 0.8	3.3 ± 0.6 ^†^
Valeric acid (mmol/kg)	2.7 ± 1.4	2.4 ± 1.2	2.7 ± 1.4
Isovaleric acid (mmol/kg)	3.0 ± 1.3	2.6 ± 1.1	3.2 ± 0.7
Total SCFAs	171 ± 35	152 ± 53	156 ± 51

Values are expressed as mean ± standard deviation. Abbreviations: STD, standard diet; HF, high-fat diet; HF + Inulin, modified HF diet containing 15% inulin; SCFAs, short-chain fatty acids. Total SCFAs is the sum of the amount of measured SCFAs. *p*-values were calculated using the *t*-test or the non-parametric Mann–Whitney U test. ^#^ *p*-value < 0.05 vs. STD; * *p*-value < 0.05 vs. HF; and ^†^ *p*-value = 0.097 vs. HF.

**Table 2:** Non-esterified eicosanoids in liver at the end of the study.

	STD	HF	HF + Inulin
5-HEPE (nmol/g tissue)	0.150 ± 0.056	0.085 ± 0.030 ^#^	0.086 ± 0.059
11-HEPE (nmol/g tissue)	0.30 ± 0.13	0.13 ± 0.05 ^#^	0.12 ± 0.06
5-HETE (nmol/g tissue)	2.0 ± 0.6	1.2 ± 0.4 ^#^	1.0 ± 0.7 ^†^
20-HETE (nmol/g tissue)	0.14 ± 0.02	0.08 ± 0.03 ^#^	0.08 ± 0.03
11(12)-EET (nmol/g tissue)	0.22 ± 0.06	0.11 ± 0.03 ^#^	0.07 ± 0.02 *
12-HETE (nmol/g tissue)	2.0 ± 0.9	1.0 ± 0.5 ^#^	0.8 ± 0.6
15-HETE (nmol/g tissue)	0.80 ± 0.28	0.33 ± 0.14 ^#^	0.29 ± 0.20
15-HETrE (nmol/g tissue)	0.41 ± 0.11	0.41 ± 0.08	0.32 ± 0.08 *
PGD_2_ (nmol/g tissue)	1.1 ± 0.4	0.32 ± 0.18 ^#^	0.33 ± 0.13
PGE_2_ (nmol/g tissue)	0.63 ± 0.28	0.23 ± 0.14 ^#^	0.31 ± 0.08 ^††^
Sum of HEPEs	0.45 ± 0.18	0.22 ± 0.07 ^#^	0.21 ± 0.11
Sum of HETEs	5.0 ± 1.7	2.6 ± 1.0 ^#^	2.2 ± 1.6 ^†^
5-HEPE/5-HETE ratio	0.076 ± 0.018	0.072 ± 0.013	0.084 ± 0.017 ^†††^
Sum of PGs	1.71 ± 0.67	0.55 ± 0.31 ^#^	0.64 ± 0.19

Values are expressed as mean ± standard deviation. Abbreviations: STD, standard diet; HF, high-fat diet; HF + Inulin, modified HF diet containing 15% inulin; 5-HEPE, 5-hydroxy-6E,8Z,11Z,14Z,17Z-eicosapentaenoic acid; 11-HEPE, 11-hydroxy-5Z,8Z,12E,14Z,17Z-eicosapentaenoic acid; 5-HETE, 5-hydroxy-6E,8Z,11Z,14Z-eicosatetraenoic acid; 20-HETE, 20-hydroxy-5Z,8Z,11Z,14Z-eicosatetraenoic acid; 11(12)-EET, 11(12)-epoxy-5Z,8Z,14Z-eicosatrienoic acid; 12-HETE, 12-hydroxy-5Z,8Z,10E,14Z-eicosatetraenoic acid; 15-HETE, 15-hydroxy-5Z,8Z,11Z,13E-eicosatetraenoic acid; 15-HETrE, 15-hydroxyicosa-8Z,11Z,13E-trienoic acid; PGD_2_, 9S,15S-dihydroxy-11-oxo-5Z,13E-prostadienoic acid; PGE_2_, 9-oxo-11R,15S-dihydroxy-5Z,13E-prostadienoic acid. Sum of HEPEs includes 5-HEPE and 11-HEPE. Sum of HETEs includes 5-HETE, 12-HETE, 15-HETE, and 20-HETE. Sum of PGs includes PGD_2_ and PGE_2_. *p*-values were calculated using the *t*-test or the non-parametric Mann–Whitney U test. ^#^ *p*-value < 0.05 vs. STD; * *p*-value < 0.05 vs. HF; and ^†^ *p*-value = 0.108, ^††^ *p*-value = 0.107, ^†††^ *p*-value = 0.077 vs. HF.

**Table 3:** Biomarkers of oxidative stress in blood at the end of the study.

	STD	HF	HF + Inulin
Plasma			
ORAC (µmol TE/mL)	15 ± 6	18 ± 6 ^#^	15 ± 5
FRAP (mmol TE/L)	0.10 ± 0.02	0.10 ± 0.01	0.10 ± 0.01 ^†^
GSH (nmol/mL)	13 ± 3	17 ± 4 ^#^	20 ± 4 *
GSSG (nmol/mL)	19 ± 5	22 ± 4 ^$^	26 ± 4 ^††^
GSSG/GSH ratio	1.5 ± 0.4	1.4 ± 0.4	1.3 ± 0.4
TBARS (nmol MDA Eq/mL)	2.2 ± 0.5	2.2 ± 0.4	1.8 ± 0.3 *
Erythrocytes			
SOD (U/g Hb)	2930 ± 630	3220 ± 440	2830 ± 350 ^†††^
CAT (mmol H_2_O_2_/min/g Hb)	29 ± 4	32 ± 4 ^$$^	34 ± 4
GPx (U/g Hb)	72 ± 12	82 ± 12 ^$$$^	92 ± 16
GR (U/g Hb)	1.2 ± 0.2	0.9 ± 0.2 ^#^	0.8 ± 0.2
TBARS (nmol MDA Eq/g Hb)	0.46 ± 0.16	0.63 ± 0.06 ^#^	0.63 ± 0.28

Values are expressed as mean ± standard deviation. Abbreviations: STD, standard diet; HF, high-fat diet; HF + Inulin, modified HF diet containing 15% inulin; ORAC, oxygen radical absorbance capacity; TE, trolox equivalent; FRAP, ferric reducing ability of plasma; GSH, reduced glutathione; GSSG, oxidized glutathione; TBARS, thiobarbituric acid-reactive substances; MDA Eq, malondialdehyde equivalent; SOD, superoxide dismutase; Hb, hemoglobin; CAT, catalase; GPx, glutathione peroxidase; GR, glutathione reductase. *p*-values were calculated using the *t*-test or the non-parametric Mann–Whitney U test. ^#^ *p*-value < 0.05 vs. STD; ^$^ *p*-value = 0.080, ^$$^ *p*-value = 0.087, ^$$$^ *p*-value = 0.100 vs. STD; * *p*-value < 0.05 vs. HF; and ^†^ *p*-value = 0.076, ^††^ *p*-value = 0.092, ^†††^ *p*-value = 0.053 vs. HF.

**Table 4:** Biomarkers of oxidative stress in tissues at the end of the study.

	STD	HF	HF + Inulin
Perigonadal adipose tissue			
SOD (U/g tissue)	64 ± 18	55 ± 20	45 ± 20
CAT (nmol H_2_O_2_/min/g tissue)	24 ± 14	25 ± 9	30 ± 7
GPx (U/g tissue)	0.69 ± 0.36	0.60 ± 0.24	0.67 ± 0.14
GR (U/g tissue)	0.34 ± 0.10	0.24 ± 0.07 ^#^	0.28 ± 0.05
GSH (nmol/g tissue)	7.0 ± 2.7	7.7 ± 3.3	8.1 ± 3.8
GSSG (nmol/g tissue)	28 ± 7	28 ± 8	28 ± 10
GSSG/GSH ratio	4.4 ± 1.6	4.1 ± 1.9	3.8 ± 3.0
TBARS (nmol MDA Eq/g tissue)	3.0 ± 1.5	1.2 ± 0.5 ^#^	0.9 ± 0.6
Liver			
SOD (U/g tissue)	5970 ± 1460	6090 ± 1270	5200 ± 1510
CAT (mmol H_2_O_2_/min/g tissue)	7.3 ± 1.6	7.6 ± 1.0	8.2 ± 1.0
GPx (U/g tissue)	42 ± 3	59 ± 5 ^#^	57 ± 6
GR (U/g tissue)	8.6 ± 2.4	7.5 ± 2.0	8.5 ± 1.8
GSH (µmol/g tissue)	1.8 ± 0.5	1.6 ± 0.2 ^$^	1.6 ± 0.4
GSSG (µmol/g tissue)	1.2 ± 0.3	1.2 ± 0.3	1.3 ± 0.4
GSSG/GSH ratio	0.75 ± 0.36	0.79 ± 0.27	0.88 ± 0.54
MDA + 4-HAE (nmol MDA Eq/g tissue)	29 ± 6	26 ± 4 ^#^	29 ± 6

Values are expressed as mean ± standard deviation. Abbreviations: STD, standard diet; HF, high-fat diet; HF + Inulin, modified HF diet containing 15% inulin; SOD, superoxide dismutase; CAT, catalase; GPx, glutathione peroxidase; GR, glutathione reductase; GSH, reduced glutathione; GSSG, oxidized glutathione; TBARS, thiobarbituric acid-reactive substances; MDA Eq, malondialdehyde equivalent; MDA + 4-HAE, malondialdehyde plus 4-hydroxyalkenals. *p*-values were calculated using the *t*-test or the non-parametric Mann–Whitney U test. ^#^ *p*-value < 0.05 vs. STD; and ^$^ *p*-value = 0.061 vs. STD.

## Data Availability

The data presented in this study are available on request from the corresponding author.

## Supplementary Materials

The following supporting information can be downloaded at: https://www.mdpi.com/article/10.3390/foods11244072/s1, Table S1: Composition of diets, Table S2: Categorization of histological parameters in perigonadal adipose tissue samples, Table S3: Categorization of histological parameters in liver samples, Table S4: Characteristics of the multiple reaction monitoring method for measurement of eicosanoids in liver.
