# Supplementary material for: Influence of Dietary Inulin on Fecal Microbiota, Cardiometabolic Risk Factors, Eicosanoids, and Oxidative Stress in Rats Fed a High-Fat Diet
Source: Foods. 2022 Dec 16;11(24):4072. doi: 10.3390/foods11244072 (PMC9778385; doi:10.3390/foods11244072)
Supplement: Supplementary file 1 [file foods-11-04072-s001.zip › foods-2061366-supplementary.pdf]

Supporting Information

Influence of Dietary Inulin on Fecal Microbiota, Cardiometabolic Risk Factors, Eicosanoids, and Oxidative Stress in Rats Fed a High-Fat Diet

Bernat Miralles-Pérez [1], Maria Rosa Nogués [1,*], Vanessa Sánchez-Martos [1], Àngels Fortuño-Mar [2], Sara Ramos-Romero [3,4], Josep L. Torres [3], Julia Ponomarenko [5], Susana Amézqueta [6] Xiang Zhang [7] and Marta Romeu [1]

[1] Functional Nutrition, Oxidation and Cardiovascular Diseases Research Group (NFOC-SALUT), Pharmacology Unit, Department of Basic Medical Sciences, Universitat Rovira i Virgili, C/ Sant Llorenç 21, E-43201 Reus, Spain.

[2] Eldine Patología, C/ Plom 32, E-43006 Tarragona, Spain.

[3] Department of Biological Chemistry, Institute of Advanced Chemistry of Catalonia (IQAC-CSIC), C/ Jordi Girona 18–26, E-08034 Barcelona, Spain.

[4] Current address: Department of Cell Biology, Physiology & Immunology, Faculty of Biology, University of Barcelona, Avd/ Diagonal 643, E-08028 Barcelona, Spain.

[5] Centre for Genomic Regulation, The Barcelona Institute of Science and Technology, Universitat Pompeu Fabra (UPF), E-08003 Barcelona, Spain.

[6] Departament d'Enginyeria Química i Química Analítica and Institut de Biomedicina (IBUB), Universitat de Barcelona, E-08028 Barcelona, Spain.

[7] Department of Chemistry, University of Louisville, 2210 S. Brook Street, Louisville, KY E-40292, USA.

* Corresponding author: mariarosa.nogues@urv.cat (M.R.N.); Tel +34-977-75-9355

Table S1. Composition of diets.

	STD[a]	HF[b]	HF + Inulin[b]
**Diet composition (g/kg feed)**			
**Carbohydrate (available)**[c]	480	457	457
Sucrose	–	340	190
Maltodextrin	–	60	60
Corn starch	–	57	57
Inulin	–	–	150
Neutral detergent fiber	180	50	50
**Fat**	40	230	230
Soybean oil	–	20	20
Milk fat	–	210	210
**Protein**	143	198	198
Casein	–	195	195
L-Cystine	–	3	3
**Mineral**	28.4	43	43
**Vitamin**	1.2	19	19
**Ash**	47	–	–
**Choline bitartrate**	1	3	3
**Macronutrients (% caloric value)**			
Protein	20.0	16.9	18.4
Fat	13.0	44.1	48.0
Carbohydrates[c]	67.0	39.0	33.7
Energy density (kcal/g)[d]	2.9	4.7	4.3

Abbreviations: STD, standard diet; HF, high-fat diet; HF + Inulin, modified HF diet containing 15% inulin. [a]Teklad Global 14% Protein Rodent Maintenance Diet (Envigo, Indianapolis, IN, USA). [b]based on TD.08811 45% kcal Fat Diet (Envigo, IN, Indianapolis, USA). [c]Carbohydrate (available) is calculated by subtracting neutral detergent fiber from total carbohydrates. [d]Energy density was calculated as estimates of metabolizable energy based on the following conversion factors: 4 kcal/g protein, 9 kcal/g fat, 4 kcal/g available carbohydrate except for inulin, which used a factor of 1.5 kcal/g inulin.

Table S2. Categorization of histological parameters in perigonadal adipose tissue samples.

Item	Score	STD	HF	HF + Inulin
Variable adipocyte diameter (%)				
Absence	0	91.7	75.0	91.7
Presence	1	8.3	25.0	8.3
Lipoblastic vacuoles (%)				
Absence	0	100	91.7	100
Presence	1	0	8.3	0
Mastocytes (%)				
Absence	0	50.0	41.7	75.0
Presence	1	50.0	58.3	25.0
Septal fibrosis (%)				
Absence	0	75.0	66.7	50.0
Presence	1	25.0	33.3	50.0
Angiomatous vascularization (%)				
Absence	0	58.3	33.3	33.3
Presence	1	41.7	66.7	66.7
Focal mild inflammation (%)				
Absence	0	100	100	100
Presence	1	–	–	–
Grade of periadipocyte histiocytes (%)				*
Absent	0	25.0	0	25.0
Mild	1	25.0	16.7	41.7
Moderate	2	16.7	41.7	25.0
Severe	3	33.3	41.7	8.3
Grade of septal histiocytes (%)				
Absent	0	100	83.3	100
Mild	1	–	16.7	–
Moderate	2	–	–	–
Severe	3	–	–	–
**Total histological score**	0–11	3 (2.25–4)	4 (3.25–4.75)#	3 (2–3)*

Values are expressed as frequencies (%) or median and 25th–75th percentiles, n = 12 rats/group. Abbreviations: STD, standard diet; HF, high-fat diet containing 34% sucrose; HF + Inulin, modified HF diet containing 19% sucrose and 15% inulin. Total histological score was the sum of evaluated items. *p*-Value was calculated by means of contingency tables using χ^2 statistics or the non-parametric Mann–Whitney U test. #*p*-Value <0.05 *vs*. STD; and **p*-Value <0.05 *vs*. HF.

Table S3. Categorization of histological parameters in liver samples.

Item	Score	STD	HF	HF + Inulin
Grade of steatosis (%)			#	*
Absence (<5%)	0	58.3	25.0	100
Mild (5–33%)	1	41.7	50.0	–
Moderate (33–66%)	2	–	25.0	–
Severe (>66%)	3	–	–	–
Steatosis localization (%)				
Centrilobular	1	–	–	–
Periportal	2	20.0	100	–
No zonal	3	–	–	–
Panacinar	4	80.0	–	–
Type of steatosis (%)				
Microsteatosis	0–100	95 (95–95)	90 (90–95)	–
Macrosteatosis	0–100	5 (5–5)	10 (5–10)	–
Lipogranuloma (%)				
Absence	0	100	91.7	100
Presence	1	–	8.3	–
Microgranuloma (%)				
Absence	0	8.3	8.3	8.3
Presence	1	91.7	91.7	91.7
Grade of portal chronic inflammation (%)			#	
Absent	0	8.3	66.7	83.3
Mild	1	83.3	33.3	16.7
Moderate	2	8.3	–	–
Severe	3	–	–	–
Grade of sinusoidal dilatation (%)			$	*
Absence	0	91.7	58.3	16.7
Mild	1	8.3	41.7	83.3
Severe	2	–	–	–
Grade of fibrosis (%)				
Absence	0	100	100	100
Portal fibrosis expansion	1	–	–	–
Incomplete porto-portal or porto-centrilobular fibrous bridges	2	–	–	–
Complete porto-portal or porto-centrilobular fibrous bridges	3	–	–	–
**Total histological score**	0–10	2.5 (2–3)	3 (1.5–4)	2 (1.25–2)*

Values are expressed as frequencies (%) or median and 25th–75th percentiles, n = 12 rats/group. Abbreviations: STD, standard diet; HF, high-fat diet containing 34% sucrose; HF + Inulin, modified HF diet containing 19% sucrose and 15% inulin. Total histological score was the sum of the following evaluated items: grade of steatosis, presence of lipogranuloma, presence of microgranuloma, grade of portal chronic inflammation and grade of sinusoidal dilatation. *p*-Value was calculated by means of contingency tables using χ^2 statistics or the non-parametric Mann–Whitney U test. #*p*-Value <0.05 *vs*. STD; $*p*-Value = 0.065 *vs*. STD; and **p*-Value <0.05 *vs*. HF.

Table S4. Characteristics of the multiple reaction monitoring method for measurement of eicosanoids in liver.

Eicosanoid	Fatty acid / Pathway	Parent (m/z)	Daughter (m/z)	RT (min)	Cone voltage (V)	Collision voltage (V)	Dwell (s)	Internal standard
5-HEPE	EPA / LOX	317.0604	114.9811	6.09	2	12	0.025	d_8-15-HETE
11-HEPE	EPA / NE	317.1881	167.1318	5.86	6	16	0.025	d_8-15-HETE
5-HETE	ARA / LOX	319.2081	114.9700	6.58	8	14	0.025	d_8-15-HETE
20-HETE	ARA / CYP	319.2081	245.2898	5.83	8	14	0.025	d_8-15-HETE
11(12)-EET	ARA / CYP	319.3996	167.1216	6.83	2	12	0.025	d_8-15-HETE
12-HETE	ARA / LOX	319.3996	179.0961	6.38	10	12	0.025	d_8-15-HETE
15-HETE	ARA / LOX	319.3996	219.1805	6.19	2	12	0.025	d_8-15-HETE
15-HETrE	DGLA / LOX	321.2819	221.2607	6.42	6	14	0.025	d_8-15-HETE
PGD_2	ARA / COX	351.1343	315.3484	3.84	2	8	0.025	d_9-PGD_2
PGE_2	ARA / COX	351.1343	315.3484	3.72	2	8	0.025	d_9-PGD_2
d_9-PGD_2		360.4043	324.2277	3.81	10	12	0.025	
d_8-15-HETE		327.2128	226.1057	6.15	2	12	0.025	
15d-PGJ_2	ARA / COX	315.1681	271.2903	5.82	2	10	0.025	d_9-PGD_2
17(18)-EpETE	EPA / CYP	317.1243	255.2942	6.21	8	10	0.025	d_8-15-HETE
18-HEPE	EPA / CYP and NE	317.1881	299.2679	5.67	4	8	0.025	d_8-15-HETE
LTB_4	ARA / LOX	335.2319	195.1517	4.98	60	14	0.025	d_9-PGD_2
LxA_4	ARA / LOX	351.2319	114.9115	4.17	26	12	0.025	d_9-PGD_2
PGD_1	DGLA / COX	353.4634	317.3532	3.85	72	10	0.025	d_9-PGD_2

Abbreviations: 5-HEPE, 5-hydroxy-6E,8Z,11Z,14Z,17Z-eicosapentaenoic acid; 11-HEPE, 11-hydroxy-5Z,8Z,12E,14Z,17Z-eicosapentaenoic acid; 5-HETE, 5-hydroxy-6E,8Z,11Z,14Z-eicosatetraenoic acid; 20-HETE, 20-hydroxy-5Z,8Z,11Z,14Z-eicosatetraenoic acid; 11(12)-EET, 11(12)-epoxy-5Z,8Z,14Z-eicosatrienoic acid; 12-HETE, 12-hydroxy-5Z,8Z,10E,14Z-eicosatetraenoic acid; 15-HETE, 15-hydroxy-5Z,8Z,11Z,13E-eicosatetraenoic acid; 15-HETrE, 15-hydroxyicosa-8Z,11Z,13E-trienoic acid; PGD_2, 9S,15S-dihydroxy-11-oxo-5Z,13E-prostadienoic acid; PGE_2, 9-oxo-11R,15S-dihydroxy-5Z,13E-prostadienoic acid; 15d-PGJ2, 11-oxo-5Z,9,12E,14E-prostatetraenoic acid; 17(18)-EpETE, 17(18)-epoxy-5Z,8Z,11Z,14Z-eicosatetraenoic acid; 18-HEPE, 18-hydroxy-5Z,8Z,11Z,14Z,16E-eicosapentaenoic acid; LTB_4, 5S,12R-dihydroxy-6Z,8E,10E,14Z-eicosatetraenoic acid; LxA_4, 5S,6R,15S-trihydroxy-7E,9E,11Z,13E-eicosatetraenoic acid; PGD_1, 9S,15S-dihydroxy-11-oxo-13E-prostaenoic acid; EPA, eicosapentaenoic acid; ARA, arachidonic acid; DGLA, dihomo-γ-linolenic acid; LOX, lipoxygenase; NE, non-enzymatic; CYP, cytochrome P450; COX, cyclooxygenase; RT, retention time.
